# Overlapping Training and Roles: An Exploration of the State of Interprofessional Practice between Behavior Analysts and School Psychologists

**DOI:** 10.1007/s40617-023-00904-y

**Published:** 2024-01-23

**Authors:** Sara M. Snyder, Heartley Huber, Tiffany Hornsby, Brian Leventhal

**Affiliations:** 1 James Madison University, 395 South High Street, MSC 6908, Harrisonburg, VA 22807 USA; 2 College of William and Mary, Williamsburg, VA USA

**Keywords:** behavior analyst, school psychologist, collaboration, training

## Abstract

Board certified behavior analysts (BCBAs) working in the schools often are charged with supporting students with and without disabilities who engage in challenging behavior. Meeting the unique needs of these students often requires a collaborative approach with other school-based professionals. We specifically sought to understand how behavior analysts engage in interprofessional collaboration with school psychologists (SPs), professionals who also have training to support students who engage in challenging behavior. We disseminated a survey across the United States targeting school-based BCBAs and SPs to further understand how their training and job responsibilities overlap. We also asked respondents about the extent to which they collaborate with the other professionals, modes of their collaboration, and barriers and facilitators in effective collaboration. Our findings in this exploratory study indicate BCBAs’ and SPs’ training and responsibilities do indeed overlap in relevant areas. Roles diverge in the frequency each professional participates in common educational and behavioral practices. Both groups report similar barriers when engaging in interprofessional collaboration, although BCBAs are more likely to report differing philosophies as a barrier. For both groups, findings suggest that BCBAs and SPs can find common ground if their professional colleagues indicate that they also value collaboration. Recommendations for future research and study limitations are also discussed.

For Board Certified Behavior Analysts (BCBAs) working in schools, addressing students’ challenging behavior is often a primary job duty. Collaboration among practitioners is well-established as a crucial component of systems, supports, and interventions to improve outcomes for students with or at-risk for disabilities in schools, including those in need of behavior support (McLeskey et al., 2017). When working with these students in schools, BCBAs often work alongside a variety of school professionals, all tasked with supporting students whose behaviors interfere with the learning environment (Brodhead, 2015). However, to best support students’ challenging behavior, effective and efficient collaboration with school personnel trained in different disciplines is essential (LaFrance et al., 2019).

Interprofessional collaboration in schools involves integrating the expertise and perspectives of professionals from different disciplines to accomplish the shared goal of providing high-quality education and care (Arora et al., 2019; McLeskey et al., 2017). Interprofessional collaboration allows for a more comprehensive view of the child in terms of identifying their strengths and needs, prioritizing concerns, and making recommendations to address co-occurring—and competing—behaviors. The importance of interprofessional collaboration is emphasized in the Behavior Analyst Certification Board’s (BACB’s) fifth edition *Task List*, which clearly describes collaboration as an essential competency required of practicing BCBAs (2017). However, in practice, collaboration among school-based professionals varies widely. On the one hand, team members from different disciplines may provide disjointed services to the student without communication or coordination. On the other hand, team members communicate frequently, participate in team problem solving, and coordinate services to provide integrated care (Bowman et al., 2021). Effective collaboration requires mutual respect and understanding of each team member’s specific discipline and expertise, and for effective collaboration to occur, individual members of an interprofessional team need to recognize that “they do not, cannot, and will not have all of the answers” (LaFrance et al., 2019, p. 710). Therefore, members of an interprofessional team must rely on the expertise of other team members to achieve optimal outcomes. This is not to say BCBAs working with other school professionals should “exchange competencies” (McWilliam, 2000, p. 63). BCBAs can practice within their scope of competence (i.e., Item 1.05; BACB, 2020) and practice services consistent with the principles of applied behavior analysis (i.e., Item 2.01). Interprofessional team members must communicate and collaborate to promote the best outcomes for clients, while practicing within their own scope of their own expertise (Bowman et al., 2021). As a result, interprofessional collaboration fosters a mutually beneficial working alliance that may contribute to more efficient problem solving for the entire system.

Although the fifth edition *Task List* (BACB, 2017)—which serves as the de facto guide for the scope and sequence of BCBA training programs—clearly describes collaboration as an essential competency required of practicing BCBAs, the extent to which BCBAs collaborate with other school professionals in practice is not well-known. Kelly and Tincani (2013) surveyed BCBAs to assess with whom they collaborated and the extent to which they collaborated. Results reflected that BCBAs often collaborate with other BCBAs, school administrators, special education teachers, and general education teachers. However, this survey did not address in what ways BCBAs collaborate with other professionals. Indeed, even less research has examined collaboration between BCBAs and school psychologists (SPs), despite potentially overlapping training and roles in schools SPs often work alongside BCBAs on interprofessional student support teams to address students’ behavioral concerns, and professionals in both disciplines may be tasked with providing behavior consultation, assessment, interventions, and trainings for school staff working directly with students. BCBAs and SPs are both trained in behavioral assessment and intervention, and although their training may differ in content, depth, and theoretical approach, their overlapping professional training offers a strong starting point for meaningful interprofessional collaboration. BCBAs may find that when they collaborate with SPs, there is potential to provide more effective and well-rounded treatments than when providing services in isolation of each other, especially when students’ cases are complex (LaFrance et al., 2019).

## Overlap in Initial Professional Preparation and Practice

The *Professional Standards from the National Association of Schools Psychologists* (NASP; 2020) and the BCBA *Handbook* (BACB, 2022) outline the similarities in degree requirements and professional standards required for SPs and BCBAs. Both disciplines are guided by national organizations that provide authored documents specifying the practitioner’s scope of practice, considerations for ethical practice, and training standards (BACB, 2017; NASP, 2020). A comparison of the BACB fifth edition *Task List* (2017) and NASP *Professional Standards* (2020) indicates clear overlap in competencies and skills related to foundations and approaches to behavior change (see Table 1). However, training programs in both disciplines are more than just the standards or competencies themselves. Given “academic freedom” at the training program level, variation in the quality, depth, and scope of instruction and fieldwork related to specific topics is expected across programs. Therefore, to better understand the extent to which their professional training supports or hinders effective collaboration between BCBAs and SPs, further examination of the overlap in their experiences in their respective training programs in terms of content, depth, and theoretical approaches is required. To date, no research has addressed this question.

**Table 1:** Overlap of Training Competencies and Skills for BCBAs and SPs

BACB 5^th^ Edition Task List	NASP 2020 Professional Standards
F-1 Review records and available data (e.g., educational, medical, historical) at the outset of the case (p. 4) H-3 Recommend intervention goals and strategies based on such factors as client preferences, supporting environments, risks, constraints, and social validity (p. 5)	Domain 4: School psychologists recognize risk and protective factors and use data and assessment to facilitate the design and delivery of curricula and interventions. . . . (p. 5)
F-6 Describe the common functions of behavior (p. 4) G-1 Use positive and negative reinforcement procedures (p. 4) G-2 Use interventions based on motivating operations and discriminative stimuli (p. 4)	Domain 4: School psychologists demonstrate skills related to behavior analysis and use systematic decision making to consider the antecedents, consequences, functions, and potential causes of behavioral difficulties that may impede learning or socialization. (p. 6)
H-2 Identify potential interventions based on assessment results and the best available scientific evidence (p. 5)	Domain 4: They [SPs] use assessment data to select and implement evidence- based mental and behavioral health interventions. (p. 6)
F-4 Conduct assessments of relevant skill strengths and deficits (p. 4)	Domain 4: They [SPs] recognize that behavioral difficulties may stem from specific skill and/ or performance deficits that can be remedied through instruction and/or reinforcement strategies. (p. 6)
H-6 Monitor client progress and treatment integrity (p. 4\5)	Domain 4: School psychologists use data to monitor academic, social, emotional, and behavioral progress; to measure student response; to evaluate the effectiveness of interventions; and to determine when to modify or change an intervention. (p. 6) Domain 4: School psychologists assist with the design and implementation of assessment procedures to determine the degree to which recommended interventions have been implemented, and they consider treatment fidelity data in all decisions that are based on intervention response and progress. (p. 6)

To help bridge the gaps between the training and professional roles of these two groups and ensure BCBAs and SPs can find common ground and develop effective interprofessional behaviors, we sought to examine the overlap in training and collaborative behaviors of BCBAs and SPs. In particular, in this exploratory study, we sought to answer the following research questions:

1. What are the training experiences and responsibilities of school-based BCBAs and SPs regarding practices and supports for students with challenging behavior?
2. What types of training do BCBAs and SPs have in interprofessional collaboration?
3. How often and in what format do school-based BCBAs and SPs collaborate?
4. What factors support or hinder interprofessional collaboration between BCBAs and SPs?

## Method

### Participants and Recruitment

After receiving institutional review board approval, we recruited a national sample of BCBAs and SPs working in schools, representing all regions of the United States, through (1) their respective national accreditation organizations and (2) social media. We distributed the survey link through NASP and the BACB. To recruit SPs, we mailed letters containing the survey link through the U.S. Postal Service to a random sample of 1,000 NASP members. We also sent an email containing a survey link to all registered BCBAs and BCBA-Ds (approximately 46,000) through the BACB mass email service. A follow-up email was sent by the BACB 2 weeks later. In addition, we shared the survey link through our personal and universities’ social media accounts and posted the link on the social media pages of regional and national professional groups. With no prior survey studies examining the collaborative practices of BCBAs and SPs to inform a power analysis, our aim was to recruit as many BCBAs and SPs as possible.

We included survey respondents who reported working primarily as a BCBA or SP in school settings. Our final sample included 276 BCBAs and 118 SPs from 40 and 35 states, respectively, representing all major regions of the United States. The BCBA sample also includes participants from Washington, DC. Table 2 (below) includes participant demographic data and their educational setting.

**Table 2:** Participant Demographics

	BCBA *(n* = 276*)*	School Psychologist *(n* = 118*)*
	*n* (%)	*n* (%)
Highest level of education		
Masters	169 (61.2)	4 (3.4)
Educational Specialist	4 (1.4)	53 (44.9)
Masters +30	84 (30.4)	36 (30.5)
Doctorate	18 (6.5)	24 (20.3)
Not reported	1 (0.3)	1 (0.9)
Years experience in profession		
Less than 1	11 (3.9)	5 (4.2)
1–5	127 (46.0)	22 (18.6)
6–10	98 (35.5)	38 (32.2)
11–15	30 (9.9)	19 (15.5)
16–20	6 (2)	16 (13.1)
21–25	0	8 (6.5)
25+^1^	5 (1.6)	11 (8.9)
Not reported	1 (0.3)	0
Years experience in schools		
Less than 1	6 (2.1)	1 (0.9)
1–5	69 (25.0)	20 (17.0)
6–10	81 (29.3)	31 (26.3)
11–15	60 (21)	26 (21.3)
16–20	37 (12.2)	16 (13.1)
21–25	13 (4.3)	12 (9.8)
25+^1^	13 (4.3)	14 (11.4)
Not reported	1 (0.3)	0
Years experience in current role		
Less than 1	14 (5.0)	8 (6.8)
1–5	177 (64.1)	31 (26.3)
6–10	53 (19.2)	38 (32.2)
11–15	23 (7.6)	15 (12.7)
16–20	7 (2.3)	12 (9.8)
21–25	1 (.3)	7 (5.8)
25+^1^	1 (.3)	8 (6.5)
Not reported	1 (0.4)	0
Current school level served^2^		
Pre-K	45 (16.3)	22 (18.6)
Elementary	125 (45.3)	62 (52.5)
Middle	75 (27.2)	32 (27.1)
High	52 (18.8)	19 (16.1)
All levels	116 (42.0)	35 (29.7)
More than one level	25 (9.1)	6 (5.1)
Not reported	0	0

^1^Due to the format of the questionnaire, we are not able to report specific years for respondents in this group.

^2^Respondents were counted twice if they reported serving more than one level or all levels, therefore percentages reported do not equal 100.

### Survey Instrument

We asked BCBAs and SPs to complete a 37-item survey using a secure, web-based platform (Qualtrics, 2020). The online survey included four major sections addressing: (1) participant demographics; (2) training in behavior support practices and collaboration; (3) general participation in common collaborative educational and behavioral practices; (4), and format, frequency, and factors affecting collaboration between BCBAs and SPs.

#### Participant Demographics

We asked participants to report their current role, highest level of education (and year of completion), total years of experience working in schools, years working in their current profession, and years of experience in their current role. We also asked the type of school setting in which they work (e.g., public, private, special education school, alternative education placement, or other), grade levels they serve (e.g., preschool/early childhood, elementary, middle/junior high, high school, or multiple levels), and their state.

#### Training in Behavior Support Practices and Collaboration

We asked participants whether they received formal training on six common behavior support practices. For each practice, respondents were asked to indicate whether they received training as part of (1) their initial coursework (e.g., required course, elective course, or as part of another course); (2) their fieldwork experience (i.e., practicum or internship); (3) professional development; or (4) some other instructional or training experience. We also asked participants how many college courses and workshops or trainings addressing interprofessional collaboration they have taken.

#### Participation in Common Collaborative Educational and Behavioral Practices

We presented 13 common collaborative educational and behavioral practices, including seven types of team-based meetings and six informal behavioral practices that SPs and BCBAs may contribute to in schools (Farmer et al., 2021). For each practice, we asked participants to rate the extent to which they personally participate or engage in each practice as part of their current role during the school year. They rated each practice using a 5-point, Likert-type scale (1 = never, 2 = once or twice per year, 3 = once or twice per quarter, 4 = once or twice per month, 5 = more than twice per month).

#### Format and Factors Affecting Collaboration

To explore their experiences with and perceptions of collaboration between BCBAs and SPs, we asked participants how often they collaborate with members of the other profession, and which formats they use to collaborate with each other (e.g., in person, phone, email, text message, video conference, other). In addition, we asked participants to select from individual- and school-level factors that may promote or hinder collaboration with members of the other profession. Participants also were asked to rate how likely they are to implement behavioral recommendations given by a colleague of the other profession, using a 4-point Likert-type scale (1 = extremely unlikely; 2 = unlikely; 3 = likely; 4 = extremely likely). Finally, we included an open-ended item asking participants to explain what practices work well for interpersonal collaboration.

### Data Analysis

We used descriptive statistics (i.e., *n*s, percentages) to summarize all BCBA and SP demographic information and ratings. To summarize participants’ scope of training on common behavioral practices, we calculated average ratings and reported percentages for respondents who received training in each format. To compare responses related to the frequency of common behavioral and collaborative practices, we conducted Mann-Whitney U tests (Mann & Whitney, 1947) to compare ratings between BCBAs and SPs. To gauge alignment among participants’ experience across format of collaboration and their perspectives on factors that support or hinder collaboration between BCBAs and school psychologists, we used Pearson Chi Square analysis.

We used thematic analysis (Braun & Clarke, 2006) to analyze responses to the open-ended question about what practices work well for interpersonal collaboration. Thematic analysis has been translated into behavioral terminology to explore practitioners’ experiences with behavioral contrast (Boyle et al., 2023). Following the six-step process, first, we read all of the responses from BCBAs and SPs. Then, we coded (i.e., paraphrased) each response and grouped responses by common themes (i.e., stimulus class). Next, we examined the responses of BCBAs and SPs to determine the most common themes across responses in each group and looked for themes common to one group, but not the other. Finally, we defined and identified examples for each theme.

## Results

**Fig. 1:**
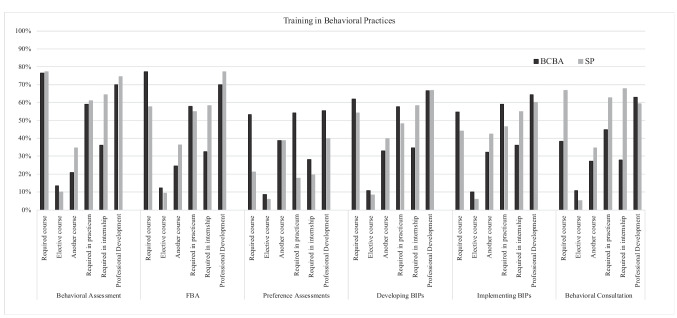
Training settings for common behavioral practices

Responses from 276 BCBAs and 118 SPs were included in this study. Results are summarized in Tables 2 through 4 and Fig. 1.

### Participant Demographics

Table 2 includes participant demographic data. Most BCBAs had a masters’ degree (61.2%) or a masters’ degree plus 30 credits (30.4%), and most SPs had an Educational Specialist degree (44.9%), masters’ degree plus 30 credits (30.5%), or doctorate (20.3%). Most BCBAs reported 0–10 years in the profession (85.4%) and had been working in their current role for 1–5 years (64.1%). In contrast, most SPs had been working in their current role for 6–10 years (32.2%) or longer (34.7%). Although our sample included BCBAs and SPs working in every school level, most SPs reported working in elementary schools. A higher percentage of BCBAs (42%) than SPs (29.7%) reported that they currently serve students across all grade levels.

### Formal Training in Behavior Support Practices and Collaboration

The scope of training in common behavior support practices is summarized for each group in Fig. 1. Overall, a higher percent of BCBAs than SPs reported receiving training in most behavioral practices. The only practice for which a higher percentage of SPs received training is behavioral consultation. A large percentage of BCBAs and SPs reported receiving preservice training (i.e., coursework and/or fieldwork) in behavioral assessments (94.6% of BCBAs and 93.2% of SPs). However, more BCBAs than SPs reported receiving preservice training in functional behavior assessment (FBA; 93.5% of BCBAs and 88.1% of SPs), preference assessments (92.4% of BCBAs and 60.2% of SPs), developing behavior intervention plans (BIPs; 92.4% of BCBAs and 60.2% of SPs), and implementing BIPs (91.7% of BCBAs and 82.2% of SPs). However, more SPs reported preservice training in behavioral consultation (77.5% of BCBAs and 93.2% of SPs). Differences in the percent of BCBAs and SPs who reported preservice training were greatest for conducting preference assessments (32.2% more BCBAs reported training) and behavioral consultation (15.68% more SPs reported training). However, these differences decreased for both practices when we calculated the percentage of respondents who received any training (e.g., preservice and/or in-service training). Only 9.8% more BCBAs reported receiving any training in preference assessments than SPs, and only 2.53% more SPs reported receiving any training in behavioral consultation. Both groups reported accessing professional development on all other common behavioral practices at similar rates. In addition, when asked how many college courses they took that addressed collaboration, SPs reported taking a significantly higher number of courses (*M* = 4.69, *SD* = 2.10) than BCBAs (*M* = 3.53, *SD* = 1.86), *F*(1, 388) = 29.04; *p* < .01.

### Participation in Common Collaborative Educational and Behavioral Practices

We asked survey respondents to report their frequency of participation in seven different collaborative educational and behavioral practices. We utilized guiding documents from NASP to develop and define some of the educational practices (e.g., IEP meetings; see NASP, 2022), 504 meetings (NASP, 2010a), and eligibility meetings (NASP 2010b). Results are summarized in Table 3. We found statistically significant differences across groups for six practices. SPs were significantly more likely to participate in eligibility meetings (i.e., initial eligibility and reevaluation), problem-solving team meetings (e.g., child study, multitiered systems of support, response-to-intervention), manifestation determination review meetings, and 504 meetings. BCBAs were significantly more likely to participate in FBA and BIP meetings. No statistically significant differences were found between BCBAs’ and SPs’ participation in individualized education program (IEP) meetings. BCBAs and SPs rated how frequently they engage in six behavioral practices (i.e., conducting behavioral assessments, conducting FBAs, conducting preference assessments, developing BIPs, implementing BIPs, and conducting behavioral consultation). BCBAs were significantly more likely to engage in all six behavioral practices than SPs.

**Table 3:** Participation in Common Collaborative and Behavioral Practices

	BCBA					School Psychologist					
	Never	Once or twice per year	Once or twice per quarter	Once or twice per month	More than twice per month	Never	Once or twice per year	Once or twice per quarter	Once or twice per month	More than twice per month	*Standardized Mann-Whitney U statistic*^*+*^
Collaborative Practices											
Eligibility meetings (initial, reevaluation)	11.4%	20.0%	21.7%	25.0%	21.0%	0.0%	1.7%	0.8%	10.2%	87.3%	11.67**
IEP meetings	1.4%	5.0%	15.5%	21.7%	55.8%	2.5%	8.5%	9.3%	12.7%	66.9%	1.46
FBA meetings	2.1%	14.4%	24.2%	22.4%	36.2%	3.4%	28.8%	41.9%	14.5%	17.1%	-4.57**
BIP meetings	1.4%	5.8%	15.9%	23.5%	52.1%	5.1%	25.5%	33.9%	16.9%	16.9%	-8.12**
Manifestation Determination Review (MDR) meetings	41.3%	38.7%	10.1%	5.4%	3.2%	31.4%	43.2%	14.4%	3.4%	7.6%	2.07*
Problem-solving team meetings	10.5%	6.8%	10.1%	19.2%	52.5%	3.4%	2.6%	7.7%	12.0%	74.4%	4.05**
504 meetings	2.1%	6.1%	10.1%	25.7%	54.3%	2.5%	8.5%	9.3%	12.7%	66.9%	8.97**
Behavioral Practices											
Conducting behavioral assessments	1.5%	6.2%	22.8%	24.3%	44.6%	6.8%	18.6%	23.7%	22.0%	28.8%	-4.20**
Conducting FBA	0.7%	14.9%	28.6%	23.9%	30.1%	17.8%	31.4%	27.1%	11.0%	12.7%	-7.10**
Conducting preference assessments	0.4%	7.3%	20.3%	25.0%	45.3%	37.3%	15.3%	22.9%	13.6%	11.0%	-10.11**
Developing BIPs	0.7%	3.6%	19.9%	30.8%	43.1%	14.4%	27.1%	29.7%	14.4%	14.4%	-9.08**
Implementing BIPs	4.0%	1.1%	5.4%	12.7%	75.0%	33.9%	20.3%	18.6%	11.9%	15.3%	-12.42**
Conducting behavioral consultation	0.7%	1.5%	1.8%	7.6%	87.3%	0.9%	13.6%	16.1%	22.9%	46.6%	-8.97**

Note: *p < .05. **p < .01. ^+^difference between U statistic and expected rank sum, divided by the expected standard deviation

### Frequency, Format, and Factors Affecting Collaboration

SPs reported significantly higher frequency of collaboration than BCBAs, *F*(1, 390) = 50.54; *p* < .01. When asked how likely they were to implement a behavioral recommendation given by a colleague of the other profession, SPs were significantly more likely to implement behavioral recommendation from a BCBA, than vice versa, *F*(1, 385) = 58.90; *p* < .01.

Both groups relied on in-person collaboration most frequently, followed by email, phone calls, video conference calls, and text messages, in descending order (see Table 4). Factors that hinder and support collaboration are addressed in Table 4. The top two factors hindering collaboration for BCBAs were not having enough time to collaborate and differing philosophies. For SPs, limited time was the most frequently selected barrier. Differing philosophies were selected significantly more by BCBAs than SPs, *X*^*2*^(1, *N* = 394) = 10.3, *p* < .01. A large percentage of SPs selected “other.” When asked to specify, 16.9% of SPs noted limited access to BCBAs (e.g., no or not enough BCBAs employed in district; BCBAs are restricted to specific programs or students). Likewise, 15.9% of BCBAs elaborated that SPs are often overextended or not often on site at their school. A smaller percentage of BCBAs (9.8%) and SPs (5.9%) also noted that their job responsibilities differ significantly, which may make regular collaboration unnecessary.

**Table 4:** Format and Factors Influencing Collaboration

	BCBA	School Psychologist	Pearson Chi Square of BCBA/ School psychologist ratings
	*n* (%)	*n* (%)	*df* = 1
Format of collaboration			
In person	253 (95.4)	90 (76.3)	17.37**
Phone call	166 (62.6)	55 (46.6)	6.15*
Email	236 (85.5)	73 (61.9)	27.31**
Text message	93 (33.7)	23 (19.5)	8.03**
Video conference	100 (36.2)	31 (26.3)	3.70
Other	13 (4.7)	5 (4.2)	0.04
Factors hindering collaboration			
Not enough time to collaborate	110 (39.9)	44 (37.3)	0.22
Differing philosophies	64 (23.1)	11 (9.3)	10.3**
School division policies/procedures do not permit collaboration	39 (14.1)	13 (11.0)	0.70
Colleagues do not value collaboration	22 (8.0)	8 (6.8)	0.17
Limited training in collaboration^1^	10 (3.6)	0 (0.0)	-
Other	109 (39.5)	57 (48.3)	2.63
Factors supporting collaboration			
Enough time to collaborate	114 (41.3)	37(31.4)	3.46
Share philosophies	133 (48.2)	69(58.5)	3.50
School division policies/procedures allow for collaboration	160 (58.0)	56(47.5)	3.69
Colleagues value collaboration	225 (81.5)	77(65.3)	12.22**
Adequate training in collaboration	153 (55.4)	75(63.6)	2.24
Other	28 (10.1)	21(17.8)	4.44*

Note: *p < .05. **p < .01.

^1^ This did not satisfy the assumptions of the Pearson Chi Square test.

When asked to indicate factors that supported collaboration, BCBAs selected colleagues who value collaboration most frequently (81.5%), and SPs indicated colleagues who value collaboration (65.3%) and adequate training in collaboration (63.6%) most frequently. However, BCBAs endorsed collaborative colleagues as a supportive factor at a significantly higher rate than SPs, *X*^*2*^(1, *N* = 394) = 12.2, *p* < .01. When asked to describe what other factors supported effective collaboration between BCBAs and SPs, both BCBAs and SPs most often indicated that access and availability of the other person and an established positive rapport improved interprofessional collaboration.

#### What Collaborative Practices Work Well?

One final open-ended question asked respondents to tell us what practices work well for interprofessional collaboration, based on the definition provided by Kelly and Tincani (2013). Ninety-one SPs and 215 BCBAs responded to the question.

For BCBAs, the most common themes across responses included having a structured process for collaboration (*n* = 85; 39.5%), designated time to collaborate (*n* = 75; 34.9%), and showing mutual respect for each other’s roles and expertise (*n* = 68; 31.6%). A smaller percent of BCBAs' recommendations for effective collaboration reflected themes of building rapport and trust for effective teamwork (*n* = 42; 19.5%), using data to guide decision making (*n* = 41; 19.1%), active listening (*n* = 28; 13.0%), sharing resources (*n* = 28; 13.0%), using approaches focused on client or stakeholder outcomes (*n* = 23; 10.7%), setting common goals (*n* = 22; 10.23%), defining roles (*n* = 17; 7.9%), and meeting face-to-face (*n* = 11; 5.1%). Less than 5% of BCBAs endorsed any of following themes in their responses: flexibility, shared decision making, additional training, more time to collaborate, commitment to evidence-based practices, shared philosophy, shared background knowledge or knowledge of applied behavior analysis, external accountability, or cultural awareness. For SPs, the most common themes across responses aligned with the top themes for BCBAs. In their responses, SPs most often included designated time to collaborate (*n* = 43; 47.3%), having a structured process for collaboration (*n* = 39; 42.9%), and showing mutual respect for each other's expertise and roles (*n* = 27; 26.7%). Less common themes included active listening (*n* = 12; 13.2%), setting common goals (*n* = 11; 12.1%), communicating clearly without judgement or jargon (*n* = 11; 12.1%), using data to make decisions (*n* = 10); 11.0%), building rapport and trust for effective teamwork (*n* = 8; 8.8%), clearly defining roles (*n* = 7; 7.7%), and shared decision making (*n* = 5; 5.9%). Less than 5% of SPs noted that approaches centered on the needs of stakeholders (i.e., students, teachers, families), shared philosophy, additional training, sharing resources, meeting in person, cultural awareness, and/or more time were important for successful collaboration. No SP responses reflected themes of shared background knowledge and understanding of applied behavior analysis, flexibility, external accountability, and commitment to evidence-based practices; fewer than 2% of BCBA responses reflected these themes.

Most responses from both BCBAs and SPs encompassed multiple themes. For example, one BCBA indicated that putting students’ needs first and “keeping egos and emotions out of decision making” is critical for effective collaboration. Similar responses from BCBAs included a focus on using objective information and research to guide decisions as a means of mitigating differences in philosophical approaches to behavior intervention. Likewise, one SP suggested that using a problem-solving model helps to mitigate differences in philosophies and centers their efforts on outcomes for the student. Across responses, clear and open communication, building rapport, and showing mutual respect for each other’s roles and expertise often appeared in the same response. Many suggested that BCBAs and SPs may struggle to understand and appreciate each other's perspectives coming from behavioral versus psychological models. As one respondent put it, BCBAs and SPs operate from their own “‘camp’ of psychology/behavior modification . . . therefore [they] have a different interpretation of the reason certain behaviors exist and, thus, the most effective means of serving a student.”

## Discussion

Although research on interprofessional collaboration in schools exists, few studies have examined the collaborative behaviors and initial and ongoing training of BCBAs and SPs. Given the overlap of professional competencies and skills defined by the BACB and NASP, which guide practitioner preparation programs, certification, and licensure, the potential for interprofessional collaboration between BCBAs and SPs to improve the effectiveness and efficiency of behavioral assessment, intervention, and evaluation for students with challenging behavior is significant. The findings from this exploratory study related to each research question yield important implications for practice and directions for future research. At the end of the discussion, we address recommendations for BCBAs working in schools alongside SPs.

### Research Question 1: What are the training experiences and responsibilities of school-based BCBAs and SPs regarding supporting students with challenging behavior?

We addressed this research question by exploring previous training and participation in common collaborative educational and behavioral practices. Overall, more BCBAs report having preservice training in more technical behavioral practices like preference assessments and functional behavior assessment, which are heavily emphasized in the fifth edition *Task List* (2017). However, a relatively large number of SPs in our sample also reported training in these more technical behavioral practices, which should not be overlooked. For example, though BCBAs were significantly more likely to receive training in development and implementation of behavior intervention plans, many SPs also reported training in this area (60.2% for developing behavior intervention plans, 82.2% for implementing), indicating a strong potential point of collaboration. However, though many SP respondents reported preservice training in behavioral assessment, our survey did not define behavioral assessment beyond the practices listed in Fig. 1. Behavioral assessment may look different for the two groups of professionals, especially considering how the two groups responded differently to questions about training on behavioral practices. Perhaps SPs typically engage in more indirect behavior assessments (e.g., questionnaires, interviews), when compared to BCBAs who may be engaging in direct behavior assessments (e.g., descriptive assessment, functional analyses). BCBAs may be specifically hired to conduct these specific practices, which may lessen SPs’ job responsibilities in behavioral assessment. BCBAs should bear in mind that their SP colleagues may also have training to conduct these behavioral practices. This common training uniquely equips SPs to collaborate with BCBAs to conduct behavioral assessment. Future research should examine more closely what specific types of behavioral assessments both groups employ in practice and investigate the most effective and efficient ways SPs and BCBAs can collaborate during the assessment process.

More SPs report preservice training in behavioral consultation, which is interesting because our BCBA respondents reported they were more likely to engage in behavioral consultation in their jobs when compared to SPs. In other words, BCBAs have less training in consultation but appear more likely to engage in behavioral consultation. This finding aligns with previous research. In their review of pretraining coursework across university programs, Shepley et al. (2017) found only 13.9% of BCBA training programs offer a course on consultation. However, our data suggests that in-service training and ongoing professional development activities may serve to bridge this gap. Many BCBA respondents reported training in conducting behavioral consultation during professional development activities. BCBAs may select these professional development opportunities because they see a need to improve their consultation skills or others (e.g., employers, continuing education providers) have identified that BCBAs need to improve skills in this area and offer professional developmental opportunities to meet this need. In practice, preservice training programs should include training on behavioral consultation, because many BCBAs are asked to do this in their professional practice. Shepley et al. (2017) found that BCBA training programs varied widely in how they prepared BCBAs to engage in consultation (e.g., through a required course). They recommended that programs preparing BCBAs to work in schools also prepare their candidates to engage in consultation, because consultation in schools is a typical format for providing behavioral services. Most respondents in both groups report receiving professional development (i.e., in-service training) in all common behavioral practices, which suggests both groups are engaged in ongoing training directly relevant to the work they do. We suspect that preservice training provides broad instruction on educational and behavioral practices, but in-service professional development may focus more specifically on processes and procedures for conducting these practices in schools. However, it is also possible that respondents more clearly recalled what they learned in professional development because it occurred more recently.

Our results indicate some deviation across groups in their participation in meetings. Both groups are likely to attend IEP meetings and do so frequently. As may be expected, BCBAs are more likely to attend FBA and BIP meetings, which aligns with their training. SPs are significantly more likely to attend problem solving meetings than BCBAs. Although the nature of some problem-solving meetings may not include behavioral concerns, BCBAs may offer valuable contributions when part or all of the discussion centers around a student’s challenging behavior, such as suggestions for assessing fidelity of existing classroom or school-wide positive behavior interventions and supports (PBIS) or recommendations for Tier 2 behavioral supports. Collaborating with BCBAs proactively during these meetings might help address challenging behaviors before they worsen and potentially reduce referrals for special education evaluations due to behavior challenges. Furthermore, including the expertise and perspectives of multiple professionals, including BCBAs and SPs, in pre-referral and early stages of the special education referral process is prudent considering the highly subjective nature of disability eligibility categories often selected for children who engage in challenging behavior (e.g., emotional disturbance) and the overidentification of students from culturally and linguistically diverse backgrounds in these categories (Sullivan, 2017). SPs overall appear to attend special education eligibility meetings more often than BCBAs, with 11.4% of BCBAs reporting having never attended an eligibility meeting. This finding is not surprising given SPs’ training and role in the special education eligibility process.

### Research Question 2: What types of training do individuals have in interprofessional collaboration?

Regarding preservice training, SPs overall reported completing more college coursework in collaboration compared to BCBAs. Kelly and Tincani (2013) found that very few BCBAs in their sample completed college coursework or in-service training on collaboration, with 67% of respondents reporting zero training in collaboration. Our results are slightly better with BCBA respondents reporting an average of 2.28 courses addressing collaboration. However, preservice training in collaboration appears to lag behind that of our SP colleagues. Although the BACB’s fourth and fifth edition *Task Lists* (2012 and 2017, respectively) and the *Ethics Code for Behavior Analysts* (2020) note the importance of collaboration, it is possible that training in interprofessional collaboration has not been fully realized at the preservice level (e.g., Kelly & Tincani, 2013; Slim & Reuter-Yuill, 2021). Future research should investigate the extent to which preservice training programs prepare future behavior analysts to collaborate with SPs and other school professionals.

### Research Question 3: In what formats and how often do school-based BCBAs and SPs collaborate?

Our findings indicate BCBAs and SPs rely on a variety of formats to collaborate. In-person collaboration was most often endorsed across both groups, followed by phone calls and emails. Professionals’ preference for synchronous communication like in-person meetings and phone calls is encouraging. Asynchronous communication modalities like email and text messaging can lead to increased miscommunication because context and emotion can be misconstrued (Byron, 2008). Miscommunication contributes to poor interprofessional collaboration (Slim et al., 2021).

This survey was initially disseminated in 2020 during the first 6 months of the COVID-19 pandemic. We asked respondents to consider the formats they typically used to collaborate prior to the start of the pandemic. Future research is needed to examine if responses have changed postpandemic. In particular, are practitioners more likely to use video conferencing postpandemic compared to the prepandemic levels reported here? When asked how often they collaborated with the other professional, SPs reported collaborating more often with BCBAs than vice versa, meaning BCBAs are more likely to work alone or with professionals other than SPs. We can also glean some information about frequency of collaboration from our data on frequency of collaborative meetings. More than half of BCBAs and SPs report they attend IEP and 504 meetings more than twice per month, signaling that there may be additional opportunities to collaborate at meetings where both are likely to attend or on shared cases, outside of formal meetings.

### Research Question 4: What factors support or hinder interprofessional collaboration between BCBAs and SPs?

BCBAs and SPs agreed that lack of time was the biggest barrier to effective collaboration. Our demographics data show that 42% of BCBA respondents worked across multiple grade levels. Given that U.S. schools typically house different school levels on different school campuses, our data suggests that many BCBAs are likely traveling to different locations in the course of their day or week. Many SPs face a similar issue. NASP’s *Professional Standards* (2020) recommends that schools staff one full-time SP for every 500 students. A recent NASP membership survey indicates that the average ratio of SPs-to-students was one SP for every 1,233 students, which far exceeds their recommendations (Goforth et al., 2021). Further, employment data for BCBAs and SPs suggest that both professionals are in high demand (BACB, 2023; Bureau of Labor Statistics, 2023). If both professional groups are stretched thin within their fields of high demand, limited time may continue to be a barrier to collaboration, especially given that both professional groups endorse in-person meetings as their most frequently used modality for collaboration. Future research should investigate if time is a barrier in schools divisions where SPs and BCBAs are assigned to the same school and report having smaller caseloads and fewer campuses to serve.

SPs reported that not having access to BCBAs was another factor that inhibited collaboration. Our findings indicating disparate participation in common educational and behavioral practices might help explain this. When SPs are primarily attending types of meetings that behavior analysts participate in less frequently, there are fewer opportunities to collaborate. In addition, working across multiple school buildings might explain why SPs experience limited access to BCBAs. As an alternative, not all school divisions employ BCBAs. Instead, BCBAs may work in schools as private contractors, or some schools may not utilize BCBAs at all. BCBAs serving as private contractors may be limited by the scope of their contract, have limited time to spend in schools, or be restricted to collaborating with certain school personnel. Any or all these restrictions could serve as barriers to collaboration. Future research is needed to better understand the nature of this reported barrier and compare the experiences of BCBAs employed by schools to those serving as contractors.

More than half the respondents in both groups report that colleagues who value collaboration are important supportive factors, although BCBAs were significantly more likely to endorse this factor as important for collaboration. This is an encouraging sign, indicating that collaboration is valued by both groups of professionals. This aligns with BCBAs’ professional values as outlined in the 2022 *Ethics Code* (BACB, 2020) and fifth edition *Task List* (BACB, 2017). If collaboration is valued, our focus should be on preparing BCBAs to emit effective collaborative behaviors, which should begin at the preservice training level. Lack of preservice training in interprofessional collaboration sets students on an early path towards operating as separate and siloed professionals (Bowman et al., 2021). Taylor et al. (2019) suggest explicit training in interpersonal skills and relationship-building may improve BCBAs’ competencies as behavior-change agents. Improvements in these skills early on also may lead to improvements in how BCBAs value collaboration and how effectively they collaborate.

BCBAs also report that differing philosophies are a barrier when collaborating with SPs. BCBAs have extensive training in principles and procedures firmly grounded in radical behaviorism. SPs and other professionals might have philosophies that include behaviorism in addition to other learning theories or approaches to child development. However, LaFrance et al. (2019) acknowledge that differing philosophies do not necessarily lead to team member incompatibility. Instead, these differing philosophies among team members can facilitate effective and comprehensive interventions focused on challenging behavior. To be effective team members, BCBAs must understand the expertise and philosophies of other professionals, and ultimately respect how they can contribute to effective outcomes for the client (Bowman et al., 2021; LaFrance et al., 2019).

In the open-ended question, BCBAs’ and SPs’ responses emphasized both designated time and a structured process to collaborate as factors that supported collaboration. Future research will need to examine what respondents mean by structured process. However, we can glean from these responses that more time might not be sufficient without some accompanying processes and procedures further outlining how SPs and BCBAs should collaborate, when, and about what.

Finally, we asked each group to endorse the following statement: How likely are you to adopt a recommendation given by the other professional? Means for both professional groups fell between *likely* and *extremely likely*. This is a promising result, because many definitions of collaboration include concepts like listening to others and sharing/exchanging ideas (D’Amour et al., 2005; Hall, 2005). Adopting a colleague’s recommendation is a step beyond sharing information. This finding suggests that members of both professional groups recognize the value of the other professionals’ expertise. This is consistent with both groups’ open-ended responses suggesting that mutual respect for roles and expertise is an important factor that supports collaboration. Further, SPs were significantly more likely to adopt a BCBA’s recommendation. This signals to the BCBA community that we might find allies and effective collaborative partners in SPs.

### Study Limitations

There are three main limitations of this study. First, we asked respondents to recall their own behavior and experiences, which could introduce inaccuracy into the data. This is particularly relevant for survey questions asking respondents to reflect upon their preservice training, which may have occurred long ago for veteran practitioners. In addition, this survey was disseminated in the first 6 months of the COVID-19 pandemic. We asked respondents to answer survey questions based on what they did prior to the onset of the pandemic, but for some participants, pandemic practices may have affected their responding, particularly regarding survey questions about frequency and modality of collaboration and educational and behavior meetings.

Second, though our sample was relatively large and representative of all U.S. regions, BCBAs responded at a higher rate than SPs, which is likely due to our limited options for recruitment for SPs (i.e., NASP supported postcard mailings rather than digital dissemination through email). A replication of this study with a larger sample of SPs may offer more insight into their collaboration with BCBAs.

Third, we did not provide definitions for behavioral and collaborative practices. Although these practices and team meetings are commonly used in schools to address challenging behaviors, we cannot determine whether respondents fully understood what each practice entails. Future research could combine survey data with record reviews and interviews to gain a more accurate picture of these collaborative practices. Researchers can use these combined methods to examine both preservice training programs and in-service practices.

### Recommendations for BCBAs Working with SPs

We echo the call of other authors who have recommended that BCBAs continue to develop and refine their effective collaboration skills (e.g., LaFrance et al., 2019; Slim & Reuter-Yuill, 2021). Our survey results were similar to that of Kelly and Tincani (2013), who found that not all preservice training programs are developing candidates’ collaboration skills. Given these findings, practicing BCBAs will need to seek professional development opportunities to enhance their collaboration skills (Slim & Reuter-Yuill, 2021), which may in turn improve outcomes for students (Taylor et al., 2019). Practitioners are encouraged to seek to understand collaboration through the lens of behavior analysis. Slim & Reuter-Yuill (2021) provide operationalized definitions of behaviors commonly associated with interprofessional collaboration, including perspective-taking and empathy.

Given their overlapping training and roles in the schools, BCBAs and SPs who combine their skill sets to collaborate together may be better positioned to effectively address complex behavior issues and maximize student outcomes (LaFrance et al., 2019). Practicing BCBAs should engage in discussion with the SPs with whom they work to better understand how their training in behavioral assessment and intervention overlaps. BCBAs and SPs must learn to respect and appreciate the other professional’s perspective and expertise, communicate using common language, and work together to accomplish mutual goals to improve student outcomes. As one participant stated, “The magic happens when each discipline has the ability to see beyond their scope of practice and truly appreciate other's perspectives.” For BCBAs seeking to improve their collaboration with SPs, Koenig and Gerenser (2006) recommend finding informal ways to interact (e.g., eat lunch with a SP) and engage in conversation about areas of overlapping training and responsibilities. Collaboration is improved when a team member (e.g., a BCBA) understands the unique philosophy and contributions that other team members bring to the collaborative process (e.g., SPs and other school professionals; LaFrance et al., 2019). From there, BCBAs should look for complementary ways to collaborate with SPs beyond formal meetings (e.g., IEP meetings). These informal collaborative experiences may improve the expediency and effectiveness of their assessment practices and improve student outcomes.

## Conclusion

To our knowledge, this is the first survey that sought to compare the training and practices of SPs and BCBAs, which also asked respondents to identify factors that support and inhibit collaboration with the other professional. Overall, our research suggests that BCBAs and SPs have training in similar areas related to challenging behavior. In practice, SPs and BCBAs have some overlapping roles and responsibilities related to supporting students, but they also attend different types of meetings and engage in different behavioral practices. Taken as a whole, our respondents indicate BCBAs and SPs want to collaborate and some are already doing so. We recommend that school-based BCBAs seek out their SP counterparts for collaboration and work towards building effective collaborative relationships. SPs’ similar yet different training may complement the work that BCBAs are doing in schools. Effective interprofessional collaboration between SPs and BCBAs may facilitate both groups meeting their shared goal of supporting the students they serve.

## Data Availability

Supporting data can be accessed by emailing the corresponding author.
